# Self-efficacy and health warnings as predictors of smoking cessation intent among Moroccan adolescents: Evidence from the Global Youth Tobacco Survey data

**DOI:** 10.18332/tid/185647

**Published:** 2024-11-15

**Authors:** Hamza Loukili, Rachid El Fatimy, Mohamed Amine

**Affiliations:** 1 Faculty of Medical Sciences, Mohammed VI Polytechnic University, Ben Guerir, Morocco; 2 Community Medicine and Public Health Department, PCIM Laboratory, School of Medicine, Cadi Ayyad University, Marrakech, Morocco

**Keywords:** smoking cessation, Moroccan adolescents, self-efficacy, health warnings, global youth tobacco survey

## Abstract

**INTRODUCTION:**

According to the literature, quitting tobacco before the age of 30 years would mitigate almost all tobacco-related hazards. In this respect, understanding behavioral patterns associated with the process of individual change to a healthier behavior is likely to contribute to tobacco control and avoidance of the related health risks, as well as to promote healthier behaviors, especially during adolescence.

**METHODS:**

This study is a secondary dataset analysis utilizing the 2016 Global Youth Tobacco Survey (GYTS) data for Morocco. Initially, a descriptive analysis is conducted to outline smoking prevalence and related behaviors among Moroccan youth, with a focus on gender differences, employing chi-squared tests for comparison. This is followed by bivariate and multivariate logistic regression analyses, which were adjusted for potential confounders to identify the determinants of intended smoking cessation.

**RESULTS:**

The survey was based on a sample of 3883 adolescents, of whom 11.07% have already tried smoking cigarettes at least once during childhood and adolescence, and 1.39% are current smokers. As for those close to the surveyed subjects, 22.2% had at least one smoking parent, and 24.1% had friends who smoked. Self-efficacy (AOR=15.54; 95% CI: 3.05–79.03) and noticing health warnings on cigarette packages (AOR=5.41; 95% CI: 2.54–11.52) were found to be important determinants of the intent to quit tobacco.

**CONCLUSIONS:**

This study is a focused analysis of self-efficacy and health warnings as predictors of smoking cessation intent among Moroccan adolescents. The study elucidates the role of self-efficacy and exposure to health warnings in shaping the intent to quit smoking among Moroccan adolescents. These findings provide evidence for developing targeted interventions that support self-efficacy and impactful health warnings to promote healthier choices and reduce tobacco use among young Moroccans.

## INTRODUCTION

Tobacco smoking is the leading preventable risk factor of Non-Communicable Diseases (NCDs), accounting for 1 in 10 deaths worldwide^1^ and an average loss of 10 years of life expectancy among smokers^2^. The alarming prevalence of smoking among adolescents and young adults, as well as the fact that the first tobacco trial occurs before adulthood^3^, call for further research aiming to explain consumption behaviors in this developmental period characterized by the adoption of risky behaviors that last throughout adulthood^4-6^, especially when the causality between cigarette smoking and NCDs is well documented^7^. Consequently, focusing on the comprehension of behavioral patterns associated with smoking cessation and promoting healthy behaviors in adolescence and young adulthood, is likely to contribute to the control of tobacco-related health hazards.

According to the literature^8-10^, the intent to perform a certain self-initiated behavioral change, such as smoking cessation, precedes the effective change. In fact, a wide array of theories tried to formalize behavioral changes, such as the Theory of Reasoned Action and the Theory of Planned Behavior, where intention and self-efficacy play a central role in performing a certain behavior^10,11^, the transtheoretical model where smoking cessation follows a pattern that includes identifiable stages, namely precontemplation, contemplation, active change, maintenance, and relapse^9,12^, the maturing-out hypothesis suggesting that change in behaviors may be due to aging and adopting the adult status^13^, and the social learning theory suggesting that behavior is acquired by conditioning and social modeling^14^.

This article attempts to identify factors associated with tobacco use cessation among adolescents aged 11–17 years in Morocco. In this perspective, we rely entirely on the Global Youth Tobacco Survey data collected by the Center for Disease Control and Prevention (CDC). A nationally representative school-based sample was constructed in a standardized manner and surveyed in 2001, 2006, 2010, and 2016. In the present study, we try to identify factors associated with tobacco use and those associated with the intent to quit tobacco smoking.

## METHODS

### Study design and setting

This study is a secondary dataset analysis of the cross-sectional Global Youth Tobacco Survey (GYTS) datasets from Morocco for the years 2001, 2006, 2010, and 2016^15^. These datasets include information across 73 variables covering a broad spectrum of tobacco usage patterns, including both smokeless tobacco products and electronic cigarettes. The GYTS, part of the Global Tobacco Surveillance System (GTSS), was developed by the WHO, CDC, and the Canadian Public Health Association to monitor tobacco use among youth and guide the implementation of effective tobacco control policies.

### Study participants and procedures

The analysis incorporates data from nationally representative two-stage samples^15,16^ consisting of 3147 adolescents aged 13–15 years for the 2001 survey, 1991 adolescents for the 2006 survey, 2106 adolescents within the same age group for the 2010 survey, and 3915 eligible participants for the 2016 survey, out of which 2948 were aged 13–15 years. The GYTS data for the case of Morocco, for the years 2001, 2006, 2010, and 2016, were retrieved from the CDC website, where all the GTSS data are available. Ethical approval for secondary analysis was not required due to the public nature of the data and the anonymization of respondents.

### Study questionnaire

The questionnaire was administered to all students who agreed to participate in each randomly selected classroom. It covered a broad range of topics related to tobacco use, including smoking behavior among adolescent and their friends and family, exposure to health warnings, and cessation attempts. Key variables included past smoking cessation attempts, self-efficacy in quitting smoking intent, exposure to advice or help for quitting, and noticing health warnings on cigarette packages. In this study, a current smoker is defined as a respondent who reported smoking at least one cigarette a day during the past 30 days. Also, intended smoking cessation was approached by reporting an affirmative answer to the question: ‘Do you want to stop smoking now?’.

In our bivariate and multivariate regression analyses, explanatory variables that we used included past smoking cessation attempts, which was approached by the question: ‘During the past 12 months, did you ever try to stop smoking?’; self-efficacy or smoker’s attitude toward their ability to stop smoking was approached by the question: ‘Do you think you would be able to stop smoking if you wanted to?’; being at the receiving end of advice or getting help in order to quit smoking was assessed using the question: ‘Have you ever received help or advice to help you stop smoking?’; and noticing health warnings and anti-tobacco messages in media and cigarette packages ascertained using the questions: ‘During the past 30 days, did you see any health warnings on cigarette packages?’ and ‘During the past 30 days, did you see or hear any anti-tobacco media messages on television, radio, internet, billboards, posters, newspapers, magazines, or movies?’.

### Statistical analysis

Descriptive analyses were first conducted to determine the prevalence of smoking behaviors. This was followed by bivariate and multivariate logistic regression analyses to identify key determinants of the intent to quit smoking among adolescents. First, a descriptive analysis was conducted to identify characteristic features in smoking prevalence and behavior among Moroccan youth. At this stage, we described our variables of interest as sample frequencies and population-weighted proportions with respect to gender, and we compared these characteristics with two-tailed chi-squared tests. Then, we performed both bivariate and multivariate logistic regression analyses to identify the determinants of intended smoking cessation among the studied population. In our analysis, odds ratios (ORs) are utilized to quantify the strength and direction of the association between smoking cessation intentions and various predictors. The 95% confidence intervals (CIs) provide a range of values within which we are 95% confident the true OR lies, offering insight into the precision of our estimates. Variables showing statistical significance at p<0.2 in the bivariate regression, were considered in the multivariate regression model^17^.

For comparison, we conducted similar bivariate logistic regression analyses for all the years other than 2016, for which the GYTS data are available for the case of Morocco, namely 2001, 2006, and 2010. All analyses conducted in this study were performed using R software (version 4.3.2).

## RESULTS

The following results are related to the 2016 survey of 3915 adolescents (Table 1), of whom 1954 (50.32%) were females, and 56.45% were aged 13–14 years at the time of the survey. Most of the respondents (82.03%) were from urban areas.

**Table 1:** Sociodemographic, smoking behavior and attitude characteristics of Moroccan adolescents, by gender, GYTS Morocco, 2016

*Characteristics*	*Female*		*Male*		*Overall*		*p*
	*n*	*%*	*n*	*%*	*n*	*%*	
**Age** (years)	1954	55.08	1929	44.92	3883	100	**0.00**
≤11	11	0.14	5	0.14	16	0.28	
12	243	5.44	180	2.24	423	7.68	
13	555	13.79	514	10.10	1069	23.88	
14	607	14.97	516	9.35	1123	24.32	
15	352	12.01	389	10.66	741	22.67	
16	144	6.83	225	8.68	369	15.51	
≥17	42	1.91	100	3.76	142	5.66	
**Residence**	1933	55.05	1914	44.95	3847	100	**0.00**
Urban	1619	39.02	1539	28.90	3158	67.92	
Rural	314	16.04	375	16.05	689	32.08	
**Pocket money per week** (Dirhams)	1947	54.95	1929	45.05	3876	100	**0.00**
Usually no pocket money	388	12.83	338	8.38	726	21.20	
≤10	622	22.05	546	16.30	1168	38.35	
11–20	469	12.09	484	11.32	953	23.41	
21–50	242	4.41	274	5.18	516	9.59	
51–100	139	2.32	134	1.68	273	3.99	
>100	87	1.27	153	2.19	240	3.46	
**Cigarette trial**	1916	55.46	1865	44.54	3781	100	**0.00**
No	1793	52.31	1574	36.62	3367	88.93	
Yes	123	3.15	291	7.92	414	11.07	
**Cigarette smoking status**	1926	55.78	1866	44.22	3792	100	**0.00**
Non-smoker	1916	55.49	1824	43.12	3740	98.61	
Smoker	10	0.30	42	1.09	52	1.39	
**Age of smoking initiation** (years)	89	24.46	250	75.54	339	100	0.52
≤7	21	6.46	45	15.12	66	21.57	
8–9	4	1.94	23	6.46	27	8.40	
10–11	10	2.51	33	7.94	43	10.45	
12–13	24	5.63	66	15.64	90	21.28	
14–15	27	6.36	68	24.10	95	30.46	
≥16	3	1.55	15	6.29	18	7.84	
**Intent to stop smoking**	40	40.43	88	59.57	128	100	**0.01**
No	27	24.38	39	30.63	66	55.01	
Yes	13	16.06	49	28.93	44.99	62	
**Attitude toward ability to stop smoking**	41	30.67	126	69.33	167	100	0.07
No	21	19.11	45	27.82	66	46.93	
Yes	20	11.56	81	41.52	101	53.07	
**Past quitting attempts**	8	31.64	107	68.36	145	100	0.27
No	22	25.11	51	38.95	73	64.07	
Yes	16	6.53	56	29.41	72	35.93	
**Smoking among parents**	1853	55.11	1812	44.89	3665	100	0.06
No	1519	43.22	1441	34.56	2960	77.78	
≥1	334	11.89	371	10.33	705	22.22	
**Friends who smoke**	1939	55.07	1917	44.93	3856	100	**0.00**
None	1610	46.94	1320	30.94	2930	77.88	
Some or most	306	7.80	553	12.75	859	20.55	
All	23	0.33	44	1.23	67	1.56	
**Smokeless tobacco use**	1921	55.65	1870	44.35	3791	100	**0.00**
No	1817	52.55	1658	38.97	3475	92.52	
Yes	104	3.10	212	5.38	316	8.48	
**Shisha smoking**	1922	55.29	1881	44.71	3803	100	**0.00**
No	1771	51.06	1616	38.49	3387	89.54	
Yes	151	4.23	265	6.22	416	10.46	

Dirhams: 1000 Moroccan Dirhams about US$99.

Table 1 also shows that a total number of 414 (10.95%) adolescents have already tried smoking cigarettes, of whom 123 (6.42%) are female, and 52 (1.37%) are current smokers. About half of the surveyed adolescents (54.57%) began smoking between the age of 12 and 15 years. As for those close to the surveyed subjects, 19.24% had at least one smoking parent, and 24.02% had friends who smoke. Using complex survey weights, the computed prevalence of cigarette smoking among Moroccan adolescents was 1.39% in 2016, and 11.07% of Moroccan adolescents have tried smoking at least once.

Regarding other tobacco products, we notice a point prevalence of hookah (shisha) smoking and smokeless tobacco use among Moroccan youth of 10.46% and 8.48%, respectively, which is unsurprisingly higher for males (6.22% for hookah smoking, and 5.38% for smokeless tobacco) than females (4.23% for hookah smoking, and 3.10% for smokeless tobacco).

According to the transtheoretical model of behavioral change, nearly 89% of current smokers were, at the time of the study, in the phase of precontemplation, as they had no intent to quit smoking. Consequently, 11% were in the contemplation phase. In addition, 36% of smoking Moroccan adolescents have been in the preparation and action phases as they tried to quit smoking in the 12 months prior to the study, but failed to maintain the new behavior and relapsed. Moreover, 53.07% showed signs of self-efficacy as they showed belief in their ability to stop smoking.

Table 2 presents the results of the bivariate regression in unadjusted odd ratios (ORs). In this setup, we used intended smoking cessation as the dependent variable, and as explanatory variables, we used past smoking attempts, attitude towards the ability to stop smoking, receiving advice from friends and family, anti-tobacco media messages, and health warnings on cigarette packages. Key outcomes suggest a significant association between smoking cessation intent and self-efficacy (OR=12.85; 95% CI: 4.83–34.20), past cessation attempts (OR=8.12; 95% CI: 3.20–20.61), and noticing health warnings on packages (OR=5.41; 95% CI: 2.54–11.52). These findings still hold when we examined the odds ratios of logistic regressions for the years 2001, 2006, and 2010, as shown in Table 3. However, odds ratios related to past cessation attempts decreased over the years, in contrast to those related to self-efficacy.

**Table 2:** Factors affecting tobacco cessation among Moroccan youth expressed in unadjusted odds ratios, bivariate regression, GYTS Morocco, 2016

*Variables*	*Overall*		*Female*		*Male*	
	*OR*	*95% CI*	*OR*	*95% CI*	*OR*	*95% CI*
Past cessation attempts	8.12***	3.20–20.61	15.99*	1.49–171.20	8.33***	2.64–26.26
Attitude toward cessation ability	12.85***	4.83–34.20	11.2*	1.75–71.63	11.85***	3.66–38.35
Receiving advice	3.75**	1.57–8.93	3.33	0.65–16.84	4.25**	1.45–12.37
Anti-tobacco media messages	2.11*	1.01–4.43	2.08	0.45–9.45	2.71*	1.09–6.76
Health warnings on cigarette packages	5.41***	2.54–11.52	3.16	0.78–12.74	5.76***	2.24–14.79

* p<0.05,

** p<0.01,

*** p<0.001.

**Table 3:** Predictors of smoking cessation among Moroccan youth, expressed in unadjusted odds ratios: bivariate regression, GYTS Morocco, 2001, 2006, 2010, and 2016

*Predictors*	*2001*		*2006*		*2010*		*2016*	
	*OR*	*95% CI*	*OR*	*95% CI*	*OR*	*95% CI*	*OR*	*95% CI*
Past cessation attempts	25.659***	9.94–66.17	16.333***	6.85–38.91	11.500***	6.73–20.74	8.125***	3.20–20.61
Self-efficacy	8.351***	3.24–21.49	4.062**	1.66–9.91	6.01***	3.41–10.57	12.857***	4.83–34.20
Receiving advice	4.100**	1.82–9.22	7.381***	2.87–18.94	7.219***	3.86–13.47	3.753**	1.57– 8.93
Anti-tobacco media messages	1.053	0.61–1.81	1.534	0.86–2.71	1.384	0.89–2.15	2.119	1.01–4.43

* p<0.05,

** p<0.01,

*** p<0.001.

Table 4 presents multivariate regression results on factors affecting tobacco cessation among Moroccan youth in 2016, with adjusted odds ratios (AORs) and 95% confidence intervals (CIs) for the whole sample, as well as female and male subsamples. Being able to stop smoking significantly increases the odds of cessation across all participants (AOR=13.84; 95% CI: 3.47–55.13), especially among male participants (AOR=17.74; 95% CI: 3.03–103.77). Noticing health warnings on cigarette packages and receiving help or advice to stop smoking, are also positively associated with cessation attempts, though the effect varies by gender. Having friends who all smoke, drastically reduces the likelihood of cessation, particularly for males, indicating a strong peer influence on smoking behavior.

**Table 4:** Factors affecting tobacco cessation among Moroccan youth, expressed in adjusted odds ratios: multivariate regression, by gender, GYTS Morocco, 2016

*Variables*	*Overall*		*Female*		*Male*	
	*AOR*	*95% CI*	*AOR*	*95% CI*	*AOR*	*95% CI*
Being able to stop smoking	13.84***	3.47–55.13	9.21	0.65–129.04	17.74**	3.03–103.77
Noticing health warnings on cigarette package	4.95*	1.42–17.23	4.72	0.47–47.29	4.33	0. 95–19.69
Receiving help/advice to stop smoking	5.24*	1.28–21.33	3.15	0 31–31.07	7.10*	1.12–44.98
Having friends who all smoke	0.02**	0.00–0.33	1	-	0.02**	0.00–0.30

AOR: adjusted odds ratio.

* p<0.05,

** p<0.01,

*** p<0.001.

## DISCUSSION

In this study, we demonstrated that attitude towards the ability to quit smoking and multiple smoking cessation attempts, as well as the influence of people close to the smoker and anti-tobacco messages, are strong factors associated with quitting behavior.

Our findings are in line with previous research on the issue. In fact, the association between the intent to quit smoking and past quitting attempts has been thoroughly investigated in both adult and adolescent populations^8,9,18^. This association was formalized by a variety of theoretical models. In this respect, the transtheoretical model, developed by DiClemente and Prochaska^12^, suggested that self-initiated quitting attempts exhibit peculiar patterns related to smoking behavioral change. This pattern is characterized by a five-stage smoking cessation process that includes precontemplation, contemplation, action, maintenance, and relapse. On the other hand, the Theory of Planned Behavior introduced by Ajzen^10^ suggested that intention to quit tobacco use immediately precedes effective quitting and translates the willingness to keep trying and allocating effort to quit smoking. Similarly, other research has aimed at testing the transtheoretical model for younger populations. As a result, the distribution of the self-initiated behavioral stages of change is significantly different for adolescents than for an older population^8^.

Correspondingly, the intent to quit smoking, in our study, was associated with self-efficacy, that is, one’s perceived ability to perform a certain behavior. Self-efficacy was documented in the case of smoking cessation by Gwaltney et al.^19^, who conducted a review of 54 studies investigating this relationship. Our findings corroborate the work of Conner and Higgins^20^, who demonstrated that implementation intentions support smoking cessation among adolescents. Similarly, Mohammadpoorasl et al.^[Bibr CIT0021]21^ identified positive attitudes towards smoking and smoker presence in the family, as significant predictors of the intention to initiate smoking among adolescents, highlighting the influence of personal and environmental factors on smoking intentions. Accordingly, our results demonstrated the significance of self-efficacy as a predictor of intended smoking cessation, as was the case in similar studies^22,23^.

The effect of the social environment was shown to be a strong predictor of the decisions related to smoking behaviors, including both smoking initiation and cessation^24-26^. The role of social influences was explored in depth by McVea et al.^27^ to determine the process of smoking cessation among youth. In that research, successful quitters were those who looked for support from others in their environment by verbalizing their intended quitting. In our study, receiving advice to stop smoking was a statistically significant determinant of intended smoking cessation. This result was quite clear in other research investigating the link between the intent to quit smoking and peer influence^5,23,28^. In fact, receiving support to quit smoking was a significant factor in smoking cessation intent among secondary school children in Hong Kong (OR=1.94; 95% CI: 1.15– 3.27)^29^. Also, Arrazola et al.^[Bibr CIT0025]25^ estimated the median point prevalence of receiving advice to quit smoking at 72.7% in a study using GYTS datasets encompassing 56 countries. Similarly, Tyc et al.^[Bibr CIT0030]30^ identified smoking among friends and parents as a strong predictor of smoking initiation among adolescent non-smokers. Also, all of the adolescent smokers in their sample reported having at least one close friend who smokes.

Health warnings in cigarette packaging (OR=5.41) and anti-tobacco media messages (OR=2.11) were also identified as determinants of smoking cessation intent. The use of targeted anti-tobacco messages in this fashion is of interest due to its wide reach among smokers and cost-efficiency^31^, and their demonstrated effect in refraining from smoking after noticing them^32^ and bridging the gap in the knowledge of health risks associated with smoking^33^.

As our analyses showed, odds ratios related to self-efficacy declined over the generations, as self-efficacy accounts for more intent to stop smoking in 2016 than in 2006. In fact, smoking adolescents from 2016 relied more on self-efficacy in their behavioral change intent and less on receiving help and being advised to stop smoking from their social environment. This particular inter-generational feature has yet to be thoroughly discussed in the literature, and the provided evidence on the matter is still conflicting. Nonetheless, some studies discuss enhanced self-efficacy among Generation Z^34,35^, which could be due to their tech literacy that assists them in the attainment of their goals. Additionally, some researchers raised the issue of narcissism among millennials and how it is correlated to self-esteem and self-efficacy^36^, which would explain the fact that adolescents from 2016 relied less on advice given by their close friends and families.

### Strengths and limitations

The study presents an examination of smoking cessation intentions among Moroccan adolescents, using the Global Youth Tobacco Survey data with a robust empirical analysis, thereby offering insights into public health and adolescent behavior research. Its strengths lie in providing a comprehensive analysis through rigorous statistical analyses, highlighting the role of self-efficacy and health warnings as predictors within the Moroccan context.

However, the study has some limitations that are inherent in research of this nature, including the reliance on self-reported data, which may introduce biases, and the cross-sectional design that precludes establishing causality. The focus on Moroccan adolescents also raises questions about the generalizability of the findings across different cultural or demographic contexts. Moreover, the study could benefit from addressing potential confounding variables to deepen the understanding of the factors influencing adolescent smoking behaviors, suggesting a need for longitudinal studies and a broader consideration of predictors to enhance the comprehensiveness and applicability of its conclusions.

## CONCLUSIONS

In this study, we analyzed the determinants of cigarette smoking cessation among Moroccan adolescents using the Global Youth Tobacco Survey dataset, based on the Theory of Planned Behavior and the Transtheoretical Model of behavioral change. This attempt was in line with behavioral change theoretical frameworks and contributes to the understanding of behavioral features of the current generation of smoking adolescents in Morocco. It provides a basis for evidence-based measures to promote healthier lifestyles among Moroccan youth as well as prevent them from consuming tobacco in its various forms.

## Data Availability

The data that support the findings of this study are available in the Centers for Disease Control and Prevention website: https://nccd.cdc.gov/GTSSDataSurveyResources/Ancillary/DataReports.aspx?CAID=2
